# Involvement of lncRNAs in cancer cells migration, invasion and metastasis: cytoskeleton and ECM crosstalk

**DOI:** 10.1186/s13046-023-02741-x

**Published:** 2023-07-18

**Authors:** Mohammad Ahmad, Louis-Bastien Weiswald, Laurent Poulain, Christophe Denoyelle, Matthieu Meryet-Figuiere

**Affiliations:** 1 (Interdisciplinary Research Unit for Cancer Prevention and Treatment), Baclesse Cancer Centre, Université de Caen Normandie Inserm Anticipe UMR 1086, Normandie Univ, Research Building, F-14000 François 3 Avenue Général Harris, BP 45026, 14 076, cedex 05 Caen, France; 2 Comprehensive Cancer Center François Baclesse, UNICANCER, Caen, France; 3 Biochemistry Division, Chemistry Department, Faculty of Science, Damanhour University, Damanhour, 14000 Egypt

**Keywords:** Long non-coding RNAs, Cancer metastasis, EMT, Cytoskeleton remodeling, Functional screening, RNA-based therapeutics

## Abstract

Cancer is the main cause of death worldwide and metastasis is a major cause of poor prognosis and cancer-associated mortality. Metastatic conversion of cancer cells is a multiplex process, including EMT through cytoskeleton remodeling and interaction with TME. Tens of thousands of putative lncRNAs have been identified, but the biological functions of most are still to be identified. However, lncRNAs have already emerged as key regulators of gene expression at transcriptional and post-transcriptional level to control gene expression in a spatio-temporal fashion. LncRNA-dependent mechanisms can control cell fates during development and their perturbed expression is associated with the onset and progression of many diseases including cancer. LncRNAs have been involved in each step of cancer cells metastasis through different modes of action. The investigation of lncRNAs different roles in cancer metastasis could possibly lead to the identification of new biomarkers and innovative cancer therapeutic options.

## Background

With the diagnosis of an estimated 19,292,789 cases and the incidence 9,958,133 deaths globally, cancer is considered the leading cause of death in 2020 [1]. Smoking, alcohol abuse and high body mass index (BMI) are the leading risk factors for risk-attributable cancer deaths and (disability-adjusted life-years) DALYs in 2019 [2]. The dissemination of cancer cells from the tissue of origin to a distant site is called cancer metastasis, which is the actual cause of death from solid cancerous diseases that are characterized by diagnosis at late stages and poor 5-year overall survival [3].

Cancer metastasis is a complex process that includes many distinct steps and signaling cascades that affect cancer cell biology [4]. Starting with extracellular signals that induce cytoskeletal remodeling affecting cellular adhesion to basement membrane and cell–cell junction, it is followed by interaction with the extracellular matrix (ECM) that allows the epithelial to mesenchymal transition (EMT) process of cancerous cells, enabling them to migrate and invade the surrounding tissue [5]. The invasion is followed by intravasation into nearby blood and lymphatic vessels that are formed by angiogenic and lymphangiogenic factors released from the tumor [6, 7]. Finally, only few cancer cells are able to survive and undergo extravasation into distant tissues and form metastasis. This occurs due to their exposure of severe stress in the blood stream through loss of adhesion to ECM, shear forces, and attacks of the immune system [8].

Furthermore, it was found that resistance to cancer therapy and metastasis shared many signaling pathways that confer metastasis-associated resistance, including chemokine receptor, Wnt/β-catenin, transforming growth factor-β (TGF-β) and receptor tyrosine kinase (RTK) signaling pathways [9]. Therefore, studying the molecular mechanisms underlying cancer metastasis are important in order to better understand and identify the primary to metastatic tumor conversion and possibly determine curative targets.

Long non-coding RNAs (lncRNAs) are a class of transcripts with more than 200 nucleotides in length and poor or absent coding capacity. Although some lncRNAs have been identified decades ago, their vast diversity was discovered recently through sequencing of full-length cDNA libraries in the human genome. The GENCODE project estimate that the human genome contains more than 16,000 lncRNA genes [10–12].

Because of their very diverse mechanisms of action, lncRNAs affect most – if not all – biological processes and it was found that lncRNAs perturbed expression may be one of the causal events of many diseases including cancer [13–15], where they are involved in metastasis-related pathways.

In this review we will underline lncRNAs critical roles in this important aspect of cancer cell biology, enlightening the need for a better understanding of their function in metastasis-related processes.

### Roles of lncRNAs in cancer

The conversion of normal cells into cancerous and tumor formation is a multistep process, through which cells acquire particular capacities that enable them to become tumorigenic. These basic hallmark capabilities, are: sustaining proliferative signaling, evading growth suppressors, resisting cell death, enabling replicative immortality, inducing angiogenesis and activating invasion and metastasis. Due to tumor microenvironment complexity additional 2 hallmarks have been added; reprogramming of energy metabolism and avoiding immune destruction [16]. Eventually, advances in the understanding of the biology of tumors have led to the emergence of four new hallmarks; unlocking phenotypic plasticity, non-mutational epigenetic reprogramming, polymorphic microbiomes and senescent cells [17].

Many studies reported alterations in lncRNAs expression in cancerous cells compared to normal ones [18–20], and through their diverse modes of action they potentially participate in each hall mark of cancer including metastasis [21–23].

### LncRNAs rely on different mechanisms of action

LncRNAs most common roles include the regulation of gene expression, at the transcriptional and post-transcriptional levels. According to their sub-cellular localization, nuclear lncRNAs can regulate gene expression by performing their function in *cis;* in the vicinity of their genomic loci of origin; or in *trans* at distal genomic loci from their site of transcription. They are also engaged at different stages of mRNA splicing. Meanwhile in cytoplasm, lncRNAs are involved in mRNA translation or stability. Interestingly, some lncRNAs can act through several different mechanisms of action [24, 25].

**Fig. 1:**
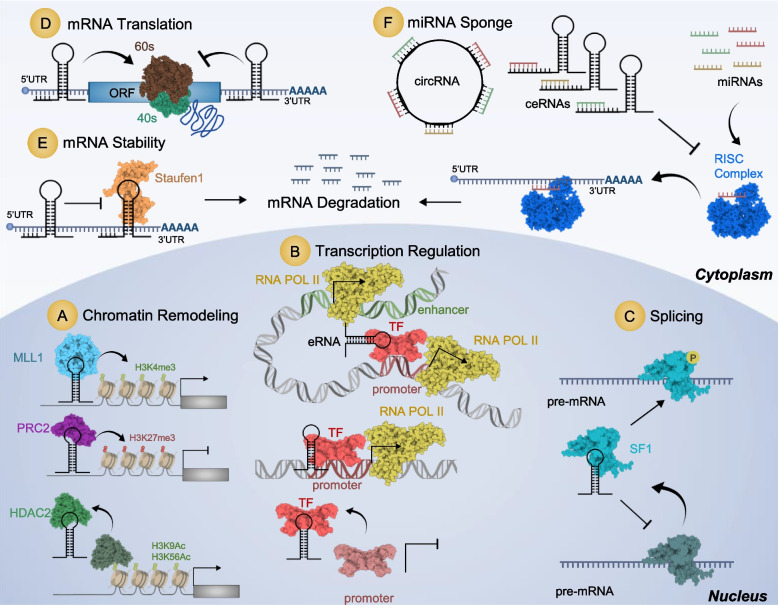
Different mechanisms of action of lncRNAs for the regulation of gene expression

In the nucleus, lncRNAs can regulate gene expression at the epigenetic level through different processes. First, they can act on chromatin remodeling by acting as guide for chromatin modifying complexes into distinct genomic loci, such as polycomb repressive complex 2 (PRC2), leading to the formation of inactive chromatin methylation modification of histone H3 at the 27^th^ lysine (H3K27me3) through its histone-methyl transferase subunit enhancer of zeste homolog 2 (EZH2). On the other hand, lncRNAs can recruit mixed-lineage leukemia (MLL) histone methyl transferase complex to gene promoters, facilitating active chromatin H3K4me3 modification, inducing gene expression [26, 27]. Also, they may act as decoy for histone deacetylases (HDACs) through direct interaction, thereby maintaining the activating chromatin modifications H3K9ac and H3K56ac (Fig. 1A) [28].

LncRNAs can also regulate gene expression at the transcriptional level through direct interaction with DNA to form R-loops. These DNA-RNA hybrids are enriched at CpG islands preventing the action of DNA methyl transferases and DNA organization in to closed nucleosomal conformation, thus induce gene expression through guiding transcription factors (TFs) to their target gene’s promoters [29, 30]. Additionally, they can act as enhancer RNAs (eRNAs) through their transcription from a gene enhancer region, and promote the formation of a chromatin loop through interaction with TFs, hence activating transcription for the genes located in the looping DNA region (Fig. 1B) [31]. LncRNAs can regulate mRNA alternative splicing at different levels, through acting as a decoy for splicing factor (SF) proteins preventing their binding to pre-mRNAs or induction of SF proteins phosphorylation promoting their target mRNA splicing (Fig. 1C) [32].

In the cytoplasm lncRNAs can affect mRNA translation, stability or turnover through binding with mRNA 5’UTR or 3’UTR to induce or repress gene expression, respectively (Fig. 1D) [33]. LncRNAs can also bind to RNA binding proteins, including both stabilizing and destabilizing factors affecting mRNA decay [34]. For instance, they can reduce mRNA stability and induce its degradation through Staufen1 mediated decay (Fig. 1E) [35].

One of the most widely studied mode of action of lncRNAs is through their action as competing endogenous RNAs (ceRNAs). Linear or circular form (circ RNA) lncRNAs can sequester miRNAs through sequence-complementarity based interaction. As a result, it prevents the formation of miRNA-induced silencing complex (RISC) and the following mRNA destabilization, hence allowing the restoration of target mRNAs expression (Fig. 1F) [36].

### Role of lncRNAs in cancer cells EMT

Epithelial to mesenchymal transition (EMT) is an important physiological process that occurs during embryo development and tissue repair and pathologically participates in disease progression, including organ fibrosis and cancer [37]. It is a multi-step process in which cells lose their cuboidal epithelial-like form and acquire a spindle-shaped mesenchymal phenotype that allows cancer cells to migrate from their original tissue and invade the neighboring circulation, enabling invasiveness and metastasis [38, 39]. Epithelial cells maintain their phenotype through different cell surface and cytoskeleton markers, such as epithelial cadherin (E-cadherin) in adherens junctions and claudin, occludin, and zonula occludens 1 in the tight junctions between adjacent epithelial cells. They also maintain cytokeratin filaments in the hemidesmosomes that anchors epithelial cells to the basement membrane to maintain their apical-basal polarity [40, 41].

In the course of EMT, cancer cells start to lose expression or function of epithelial markers, such as E-cadherin and gain mesenchymal markers, such as vimentin, mesenchymal neural cadherin (N-cadherin), integrins (α2β1, α5β1), collagens (Type (I) and (II)), fibronectin and matrix metalloproteinases (MMPs 1, 3 and 9) [42, 43].

Vimentin is an intermediate filament protein and responsible for cellular motility through interaction with motor proteins for trafficking of cellular organelles, as well as, targeting mesenchymal proteins towards membranes [44]. N-cadherin connects with cytoskeleton through α-catenin, β-catenin, p120 catenin, and stabilizes RTKs increasing their signaling and induction of EMT [45]. Integrin α2β1 interacts with type (I) collagen in ECM through the action of ECM remodeling MMPs and promotes the dissociation of E-cadherin and nuclear translocation of the transcription factor β-catenin [46]. Integrin α5β1 increases cellular adhesion toward ECM protein fibronectin inducing cellular migration [47].

Wnt/ β-catenin and RTK signaling, together with TGF-β, Notch or Hedgehog and among the many signaling pathways involved in EMT [48, 49]. Through these signaling pathways different TFs have been integrated in the repression of epithelial genes and induction of mesenchymal ones, such as Snail family: SNAIL1 and SLUG (also known as SNAIL2); zinc-finger E-box-binding (ZEB): ZEB1/2 and basic helix–loop–helix (TWIST1/2) [50, 51].

Numerous findings identified the principal role of lncRNAs in cancer metastasis by modulating EMT and migration/invasion processes (Table 1).

**Table 1:** Examples of metastasis-associated lncRNAs

LncRNA	Function	Mechanism of action	Cancer Type	Reference
PTAR	Increases EMT and metastasis	Represses miR-101-3p resulting in ZEB1 upregulation	OC	[52]
H19	Induces metastasis	TGF-β-induced expression resulting in upregulation of SLUG	Different types	[53]
MEG8	Induces EMT	Interacts with EZH2 and represses miRNA-34a and miRNA-203 genes, leading to SNAIL1/2 upregulation	LC & PCa	[54]
HOXD-AS1	Induces migration and invasion	Suppresses miR-130a-3p resulting in SOX4 upregulation and concomitant EZH2 and MMP2 expression	HCC	[55]
ATB	Increases metastasis	Suppresses miR-200 family resulting in increased ZEB1 and TWIST1 expression	BC	[56]
N-BLR	Induces migration and invasion	Targets miR-200c-3p resulting in upregulation of N-cadherin, SNAIL and ZEB1	GC	[57]
	Induces migration and invasion	Targets miR-200c-3p and miR-141-3p	CRC	[58]
MALAT1	Induces proliferation and metastasis	Suppresses miR-200a resulting in increased ZEB1 expression	LC	[59]
CRYBG3	Inhibits proliferation, migration and invasion	Interacts with G-actin preventing its polymerization to F-actin	LC	[60]
LINC00857	Induces proliferation and metastasis	Suppresses miR-103b resulting in upregulation of RhoA	PCa	[61]
LCAT1	Induces metastasis	Suppresses miR-4715-5p resulting in upregulation of Rac1	LC	[62]
DANCR	Induces metastasis	Suppresses miR-27a-3p resulting in upregulation of LIMK1	HCC	[63]
H19	Induces proliferation, migration and invasion	Suppresses miR-15b resulting in upregulation of Cdc42	HCC	[64]
LINC00452	Induces migration and invasion	Suppresses miR-501-3p resulting in upregulation of ROCK1	OC	[65]
ZFAS1	Induces metastasis	Suppresses miR-3924 resulting in upregulation of ROCK2	Pancreatic adenocarcinoma	[66]
H19	Inhibits migration	Suppresses TGFβI through its derived miR-675	Prostate cancer	[67]
HOTAIR	Induces metastasis	Col-1 induces HOTAIR expression	NSCLC	[68]
Gm26809	Induces proliferation and migration	Reprogramming of normal fibroblasts into CAFs	Melanoma	[69]
LINC00092	Induces metastasis	Interacts with PFKFB2 resulting in increased glycolysis and sustained CAFs features	OC	[70]
NAS1	Induces dormancy of DTCs	Interacts with NRF2 mRNA-5’UTR resulting in upregulation of NRF2 and ΔNp63 down regulation	BC	[71]

Bioinformatic analysis of ceRNA network in mesenchymal ovarian cancer (OC) identified lncRNA pro-transition associated RNA (PTAR). PTAR-associated upregulation of ZEB1 EMT-associated TF was observed in mesenchymal sub-types as compared to epithelial sub-types in The Cancer Genome Atlas (TCGA) OC data sets. Moreover, PTAR overexpression increased EMT and metastasis of OC in vitro*,* while PTAR knockdown (KD) diminished OC tumorigenicity and metastasis in vivo. Functional investigation identified how PTAR controls ZEB1 levels. PTAR acts as ceRNA for miR-101-3p, that directly targets ZEB1 mRNA; PTAR upregulation thus leading to increased ZEB1 levels [52].

LncRNA H19 was observed to be highly expressed in different metastatic tissues, regardless of the primary tumor origin. TGF-β-induced expression of H19 leads to the upregulation of SLUG TF that in turn upregulates H19 expression in a positive feed-back loop. The resulting inhibition of E-cadherin induces metastasis in cancer cells of several origins [53].

LncRNA maternally expressed 8 (MEG8) is related to TGF-β-mediated EMT of both lung cancer (LC) and pancreatic cancer (PCa) cell lines. MEG8 recruits of EZH2, a member of the PRC2 repressive complex, to miRNA-34a and miRNA-203 genes promoter regions. The subsequent inhibition of the expression of these miRNAs leads to SNAIL1/2 upregulation, repression of E-cadherin, and promotes EMT [54].

The upregulation of lncRNA HOXD-AS1 was associated with migration and invasion of hepatocellular carcinoma (HCC) cells in vitro and distant lung metastasis and poor prognosis in vivo. It was shown that STAT3-mediated HOXD-AS1 overexpression induces SRY-related HMG-box 4 (SOX4). HOXD-AS1 competitive binds miR-130a-3p thus preventing SOX4 miRNA-mediated degradation [55]. SOX4 has been upregulated in numerous cancers and associated with TGF-β-mediated EMT and metastasis [72].

LncRNA activated by TGF-β (ATB) is highly expressed in breast cancer (BC) patients and correlated with increased metastasis and decreased overall survival. It was found that lncRNA ATB act as ceRNA for several miRNAs of the miR-200 family, namely miR-200a/b/c, miR-141 and miR-429. Through these interactions, ATB can increase ZEB1 and TWIST1 expression, these transcription factors promoting in turn vimentin expression and BC cells migration and invasion. [56].

LncRNA N-BLR has high expression levels in gastric cancer (GC) tissue compared to normal gastric tissue. Downregulation of lncRNA N-BLR reduced the migration and invasion abilities of GC cells. Mechanistic characterization identified that N-BLR induced EMT through targeting miR-200c-3p. miR-200c-3p has anti-EMT characteristics through down regulation of N-cadherin as well as SNAIL and ZEB1 TFs [57]. Moreover, N-BLR regulates colorectal cancer (CRC) metastasis through sponging the anti-metastatic miRNAs miR-200c-3p and miR-141-3p, and its KD inhibits CRC cells migration and invasion [58].

Furthermore, the lncRNA metastasis-associated lung adenocarcinoma transcript 1 (MALAT1), upregulated in various types of cancers [73], promotes LC proliferation and metastasis by acting as ceRNA for anti-metastatic miR-200a, inducing ZEB1 TF expression [59].

Altogether, it is interesting to note that ATB, N-BLR and MALAT1 lncRNAs are all acting on EMT through the same mode of action, by ceRNA relationships with miRNAs from the mir-200 family. This miRNA family have broad roles in EMT, metastasis ECM remodeling and overall appear as master regulators in most of metastasis-related processes [74]. Hence, in this regard, lncRNAs seem to appear in turn as an efficient and specific tool to control the activity of miRNAs in a post-transcriptional manner, adding to the necessary complexity to fine-tune gene expression.

### Role of lncRNAs in cancer cells cytoskeleton remodeling

Cytoskeleton is a complex network of filamentous proteins that maintain cellular architecture and interaction between these proteins is crucial for cytoskeleton function. Cytoskeleton is composed of three main components; (i) Actin microfilaments; for cell morphology maintenance and locomotion, (ii) intermediate filaments which are cell type-specific and made up of vimentin and keratins, and (iii) microtubules including α- and β-tubulins that serve as support for intracellular organelles and segregation of chromosomes in the cell cycle [75, 76].

Actin cytoskeleton remodeling is a key characteristic in the eukaryotic cell to perform different functions, including cell motility, cytokinesis, membrane trafficking, and endocytosis. Actin filaments dynamics come from its ability to switch between monomeric globular (G) form and polymeric filamentous (F) form. During actin polymerization, ATP-G-actin monomers are added to the fast-growing barbed end of actin filaments and experience structural transition into flattened (F) actin form that converts ATP into ADP with its ATPase activity during which added to the slowly-growing actin filament tapered ends. This process is regulated by actin binding proteins, which are implicated in assembly, disassembly, capping and crosslinking of actin filaments, such as actin monomer binding protein profilin (PFN1), cleavage protein cofilin (CFL1) and capping/ branching actin related protein (Arp2/3) complex [77–79].

In case of normal physiological condition with low CFL1/actin ratio, CFL1 binds ADP-F actin from the elongated pointed ends causing its release, then PFN1 replaces ADP with ATP creating a new pool of G-actin that can be added to the barbed end. On the other hand, in cancer cells with high CFL1/actin ratio, CFL1 binds rapidly to F-actin, causing its saturation and stabilization into twisted form that allow its separation as CFL1-saturated actin bundles from the pointed ends. CFL1 rapidly dissociates from actin, causing the emergence of new highly-growing barbed ends which in turn induces cellular motility [80–83]. The phosphorylation/ dephosphorylation status of CFL1 ascertain its activity, as CFL1 phosphorylation inhibits its activity and ability to bind F-actin and hence actin filaments remodeling [84]. Moreover, CFL1 upregulation has been perceived in many cancers, since it induces metastasis and inhibits apoptosis of cancer cells [85–87].

The Arp2/3 complex responsible for actin branching through nucleation of a new growing filament from the nascent filament or capping of the pointed end. This branching process will produce heavily populated branched actin filaments [88].

The spatially growing actin filaments against plasma membrane develop filopodia, lamellipodia and invadopodia which are subcellular protrusions that allow cancer cells to invade the extracellular niche during metastasis [89]. The ras homolog family (Rho) GTPase–dependent signaling cascades regulate filopodia, lamellipodia and invadopodia formation. There are three main factors from the Rho GTPases considered as key cytoskeleton regulators; Rho (A, B, and C), Rac (1,2, and 3) and cell division cycle 42 (Cdc42) [90]. RhoA is found in cell membranes and regulates the formation of actin-myosin bundles, stress fibers, focal adhesions and lamellipodia. RhoB is found in the endosomes, while RhoC regulates the phagosomes [91].

Rac1 is mainly present in the membrane and is responsible for the development of lamellipodia and invadopodia, while Rac2 induces cellular adhesion to intercellular adhesion molecule-1 (ICAM-1). Rac3 is responsible for the adhesion of invadopodia to the ECM allowing its degradation [92]. Cdc42 is a stimulator of filopodia formation and responsible for cancer cells migration and invasion to the ECM [93]. These Rho GTPases exert their action through downstream effector Rho-associated coiled-coil containing protein serine/threonine kinase (ROCK) family of proteins including ROCK1 and ROCK2 [94, 95].

LncRNAs have shown a great influence on actin cytoskeleton remodeling in cancer not merely by direct interaction with actin and its related proteins but also its regulatory pathways including Rho/ROCK signaling pathway (Table 1) [96].

LncRNA CRYBG3 inhibits proliferation, migration and invasion of LC cells through direct interaction with G-actin preventing its polymerization to F-actin. As a result, CRYBG3 blocks LC cells in M phase of the cell cycle leading to the generation of bi-nucleated cells and eventually apoptosis [60].

High expression of LINC00857 was associated with PCa advanced stage and metastasis. LINC00857 promotes PCa cells proliferation and metastasis through the regulation of miR-103b/RhoA axis, by acting as ceRNA for miR-103b that targets RhoA mRNA. LINC00857 overexpression thus leads to an increase in RhoA expression, favoring metastasis [61].

The newly discovered lncRNA lung cancer associated transcript 1 (LCAT1) was shown to be upregulated in LC tissues. It has been shown that LCAT1 is a ceRNA to miR-4715-5p, which is targeting Rac1 mRNA. It was thus shown that, by decreasing Rac1 expression levels, LCAT1 KD inhibits LC metastasis in xenograft mouse models [62].

The differentiation antagonizing nonprotein coding RNA (DANCR) is highly expressed in HCC cells. DANCR induces HCC cells metastasis by acting as a ceRNA for miR-27a-3p, therefore upregulating the expression of its direct target LIM domain kinase (LIMK1) mRNA. LIMK1 is substrate of ROCK1 and responsible for CFL1 phosphorylation and hence its inactivation, therefore controlling EMT [63].

LncRNA H19 induces HCC cells proliferation, migration and invasion, while its KD promotes HCC cells apoptosis. Mechanistically, H19 is a ceRNA to miR-15b preventing its binding to Cdc42 mRNA 3’UTR eventually promoting Cdc42/PAK1 signaling pathway [64]. PAK1 (p21-activated kinase 1) has been shown to be upregulated in many cancers [97] and is activated through phosphorylation by Cdc42 and activates LIMK1 [98].

The lncRNA LINC00452 is upregulated in both OC cells and tumor tissues in patients and negatively associated with relapse-free survival of OC patients. LINC00542 induces OC cells migration and invasion by acting as ceRNA for miR-501-3p and subsequent derepression of ROCK1 expression [65].

The lncRNA ZNFX1 antisense RNA 1 (ZFAS1) is transcribed in antisense orientation of zinc finger NFX1‐type containing 1 (ZNFX1). It is abnormally expressed in many cancers [99]. LncRNA ZFAS1 influences pancreatic adenocarcinoma metastasis through RhoA/ROCK2 signaling pathway by functioning as ceRNA for miR-3924, which itself inhibits ROCK2 expression [66].

### Role of lncRNAs in cancer cells ECM regulation

ECM has a crucial role for all biological processes through support of tissue architecture, cell adhesion anchor, reserve of water and different growth factors, as well as control of several intracellular signaling pathways [100]. ECM is an assorted fabric, constructed from proteins (mainly collagen, fibronectin, elastin and laminin), glycosaminoglycans (i.e. chondroitin sulfate, heparin sulfate and hyaluronic acid), proteoglycans (i.e. hyalectans, aggrecan, versican and decorin), and ECM modifying enzymes (i.e. ADAM, ADAMTS and cathepsin). ECM differs from organ to organ in composition [101].

ECM is considered a main component of tumor microenvironment (TME) beside cancer-associated fibroblasts (CAFs), immune cells, endothelial cells and pericytes [102]. CAFs are the main source of ECM components along with the tumor cells that produce notable amount of ECM during cancer development [103]. Alterations in ECM composition such as cytokines and MMPs, and excessive collagen accumulation (mainly type (I)) are involved in cancer cells proliferation and metastasis [104]. These ECM modifications depend on cancer progression stage [105].

During the early stage of cancer formation cancer cells excessive secretion of TGFβ induces fibroblasts recruitment and activation into CAFs. This leads to diminished production of MMPs that allow increased ECM fibers deposition and tumor stiffness [106]. Afterwards, the signaling from ECM induced EMT of cancer cells that secrete MMPs enabling cancer cells invasion to ECM niche [107, 108]. Type (I) collagen (Col-1) is highly enriched in TME favoring tumor progression [109]. Col-1 binds to α1β1 and α2β1 integrins on cancer cells plasma membrane and inhibits cAMP-dependent protein kinase A, resulting in the actin cytoskeleton remodeling and EMT [110].

There are lncRNAs involved in cancer cells-ECM crosstalk that allow metastatic dissemination of cancer cells into nearest blood vessels (Table 1) [111].

It has been found that lncRNA H19 and its derived miR-675 are downregulated in metastatic compared to non-metastatic prostate cancer cell lines, while H19 upregulation increased miR-675 levels and inhibits metastatic cells migration. Mechanistic investigation identified that H19 affects ECM as miR-675 targets 3’UTR of transforming growth factor β induced protein (TGFβI), inhibiting its translation [67]. TGFβI is an ECM protein that showed dual function as tumor promoting and suppressive factor. In several studies it has been shown that TGFβI upregulation is associated with cancer cells invasion, metastasis and extravasation [112].

The elevated expression of lncRNA HOX Transcript Antisense RNA (HOTAIR) is linked to lymph node metastasis and poor survival in patients with lung adenocarcinoma and squamous cell carcinoma, and is responsible for brain metastasis in non-small cell lung cancer (NSCLC) [113]. HOTAIR was up-regulated in NSCLC cells in a 3D culture model supplemented with Col-1. Col-1 induced the expression of a reporter gene controlled by HOTAIR promoter, while HOTAIR expression could be reduced by using antibody against Col-1 receptor α2β1 integrin, indicating the role of lncRNAs in the cancer cells-ECM crosstalk [68].

Exosomes are double-membraned vesicles secreted by different types of cells and can carry various types of cargoes such as lncRNA, mRNA, miRNA, lipids or proteins. Exosomes derived from cancer cells are involved in TME modulation and induce tumor cells migration and invasion [114]. It was found that melanoma-derived exosomes containing lncRNA Gm26809 induced melanoma cells proliferation and migration through reprogramming of normal fibroblasts into CAFs, while this effect was revoked through lncRNA Gm26809 KD in melanoma cells [69].

Elevated levels of the prometastatic chemokine CXCL14 in CAFs has been associated with poor prognosis in OC. It was found that high CAFs-associated CXCL14 levels induced upregulation of lncRNA LINC00092 in OC cells. Mechanistically, LINC00092 binds to the glycolytic enzyme 6-phosphofructo-2-kinase/fructose-2,6-biphosphatase 2 (PFKFB2). This results in an alteration in glycolysis which supports CAF metastasis-promoting functions [70].

### Role of lncRNAs in metastatic cancer cells dormancy and reactivation

Finally, in the early metastasis, disseminated tumor cells (DTCs) to distant organs is undergoing a dormancy stage, in which DTCs harbor reduced proliferation accompanied by sustained survival for years before reactivation for proliferative metastasis. This dormancy stage of DTCs is occurring together with immune evasion and acquisition of high plasticity characteristics [115]. Suggesting evidences identify the role of some lncRNAs in DTCs dormancy and reactivation in distant metastatic organs (Table.1).

It was found that the lncRNA NR2F1-AS1 (NAS1) was upregulated in dormant mesenchymal-like BC stem-like cells (BCSCs) as compared to epithelial-like BCSCs. Mechanistic investigation identified that NAS1 binds to the GC-rich region in 5′UTR of NR2F1 mRNA. This leads to the recruitment of the RNA-binding protein PTBP1 to promote internal ribosome entry site (IRES)-mediated NR2F1 translation. As a result, this suppresses the expression of *TP63* gene variant *ΔNp63* and hence the expression of miR-205, which is transcriptionally regulated by *ΔNp63.* Because miR-205 is known to maintain epithelial features and repress EMT through targeting ZEB1, NAS1 ultimately favors EMT in dormant cells [71].

### Identification and functional characterization of lncRNAs

Although lncRNAs have differential expression patterns between normal and cancer tissues, it is insufficient to identify the role of lncRNAs as tumor initiator, promoter or suppressor [116]. Due to their lack of open reading frame (ORF), their unique spatio-temporal fashion of expression and their numerous modes of action, attributing their function is challenging [117]. Therefore, functional screening of lncRNAs constitutes an interesting approach to identify their functions and potential cancer curative targets.

Different functional screening approaches have been used, such as RNA interference (RNAi), and antisense oligo nucleotides (ASOs) for post-transcription targeting of lncRNAs. In addition, there are high-throughput approaches for concurrent screening of thousands of lncRNAs, including clustered regularly interspaced short palindromic repeats (CRISPR)/CRISPR-associated protein 9 (Cas9) functional screening systems that targets lncRNAs on both genetic, and epigenetic levels [118].

#### RNA interference (RNAi)

RNAi or also known as post transcription gene silencing (PTGS), is a biological process that occurs due to the introduction of double-stranded small interfering RNA (siRNA) molecules into the cellular system. The siRNAs are short double-stranded 21 bp RNA molecules that directs the RISC complex to its cellular RNA targets, resulting in its degradation by Argonaute 2 (Ago2) protein [119–121].siRNAs are easily to be generated and delivered in to target cells through transfection or electroporation. They can be applied as a pool of different siRNAs, or chemically modified siRNAs in order to reduce off-target effects [122]. The RNAi screening provides an efficient tool for finding of genes related to specific pathway, structure or function through combination of gene KD and its mutant phenotype [123].

The high-content RNAi screening targeting more than 2000 lncRNAs in HeLa cells identify several lncRNAs implicated in cell cycle crucial steps including chromosome segregation, mitotic duration and cytokinesis. The lncRNA *linc00899* has been identified to control microtubule dynamics and hence, mitosis in different cell types. Mechanistic investigation identified that lncRNA *linc00899* mediates the transcriptional repression of the tubulin polymerisation-promoting protein TPPP/p25. It was found that overexpression of TPPP increases tubulin acetylation and also microtubule stability via microtubule bundling and *linc00899-*depleted cells showed altered microtubule dynamics and delayed mitosis [124].

#### Antisense Oligonucleotides (ASOs)

ASOs are 12–25 nucleotide single-stranded chemically modified oligonucleotides that mediate RNAse H degradation of target RNA. RNAse H is a ubiquitous enzyme that cleave RNA in DNA-RNA duplex [125]. ASOs chemical modifications allow them to be easily delivered, active in both cytoplasm and nucleus and prevent their degradation by endonucleases and exonucleases [126].

As a part of FANTOM 6 project, which used antisense ASOs to KD 285 lncRNAs in primary human dermal fibroblasts (HDF) associated with molecular phenotyping using CAGE-seq, identified several lncRNAs associated with cell cycle defects, further supporting the role of lncRNAs in cell cycle progression. It was found that ZNF213-AS1 regulates HDF cells growth, migration, and proliferation [127].

However, ASO-mediated KD of lncRNA transcript may activates premature transcription termination, since ASOs can work on the nascent lncRNA transcript inducing its cleavage during the poly adenylation process resulting in degradation of the residual RNA polymerase II (Pol II)-associated RNA in XRN2-dependent manner. On the other hand, targeting the transcript 3’end with ASOs escapes the premature.

Transcription termination, therefore, the effect on transcription must be studied for proper use of ASOs on both experimental and therapeutic levels [128].

#### CRISPR/Cas9 System

Using artificial single chain guide RNA (sgRNA) and recombinant *Streptococcus pyogenes* Cas9, the targeted genome engineering of human cells become possible [129, 130].

The action of sgRNA/Cas9 complex resulting in DNA double strand break (DSB) that can be further repaired by DSB repair pathways. The two major DSB repair pathways are template-dependent error-free homologous recombination (HR) and template-independent error-prone non-homologous end joining (NHEJ). NHEJ resulting in insertion/deletion (indel) mutations in the genomic DNA that induce gene KO, while HR can be used for gene substitution or gene knock-in (KI) through addition of DNA template [131, 132].

Moreover, nuclease-null or dead Cas9 (dCas9) can be used as a precise tool of epigenetic regulation of gene expression [133]. dCas9 can be coupled to transcription repressor domain in CRISPR-interference (CRISPRi) system, such as Krüppel-associated box (KRAB) to inhibit transcription of multiple endogenous genes [134]. KRAB is a naturally-occurring transcriptional repression domain involved in recruitment of heterochromatin-forming complex that induces histone methylation and deacetylation [135]. On the other hand, dCas9 fused with transcription activator domain in CRISPR-activation (CRISPRa) system, such as the catalytic core of human histone acetylase p300, can activate gene expression from both promoters and enhancers [136].

Pooled CRISPR screening introduces numerous parallel genetic mutations into a pool of cells [137]. Pooled screening started with the design of genome-wide gRNA library to target hundreds to thousands of genes followed by a specific biological challenge, such as resistance to anticancer drug. Viruses are usually used for transfection and applied at low titres (multiplicity of infection, MOI, ~ 0.3), so that each cell can harbor one genetic perturbation. Then, enabling cells to grow under anticancer treatment allows the evaluation of phenotypic changes following the CRISPR-induced genetic perturbations through parallel sequencing of gRNAs [138–141]. This sequencing-based counting of gRNAs will identify those enriched or depleted after treatment. The CRISPR pooled screening identifies rated list of genes involved in the phenotype of interest [142].

A CRISPR/Cas9 genome-wide functional screening of lncRNAs has been applied, allowing the screening of 10,996 lncRNAs and the identification of 230 lncRNAs that are essential for cellular growth of chronic myeloid leukemia cells [143]. Additionally, using CRISPR/Cas9 library for genome-scale deletion of lncRNAs allow the identification of 51 lncRNAs that positively or negatively regulate HCC and HeLa cells growth and metastasis [144].

### RNA-based cancer therapeutics

There are three siRNA drugs have been approved by FDA from 2018 to 2020 (patisiran, givosiran, and lumasiran) and seven other siRNA candidates in Phase III clinical trials (vutrisiran, nedosiran, inclisiran, fitusiran, teprasiran, cosdosiran, and tivanisiran). These siRNA drugs are indicated for non-cancerous rare or orphan diseases, whose patients have an urgent need for novel and effective therapies [145]. Also, there are variety of siRNA-based cancer therapeutics are in the early clinical trial stage. However, there are many challenges to siRNA drug development including; site-specific delivery, endosome trapping and risk of activation of an undesired immunogenic response [146]. Moreover, cancer is not a one gene disorder but a multifactor illness and siRNAs showed off target effects through incomplete base pairing of seed region with undesired target genes’ transcripts and that may lead to identification of false druggable targets and off-target toxicity of cancer drugs in clinical trials [147–149].

Due to lack of information for the tertiary structure for RNA molecules, including lncRNAs, ASOs represent an efficient tool to target lncRNAs based on the sequence alone in the pre-clinical studies [150]. The novel lncRNA AC104041.1 is overexpressed in head and neck squamous carcinoma (HNSCC), enhance tumor growth and metastasis in vitro and in vivo, and associated with poor survival of HNSCC patients. Using ASOs targeting AC104041.1 enhances salinomycin treatment efficacy in both HNSCC cells and patient-derived xenograft (PDX) models. Salinomycin is a highly effective antibiotic that eradicate cancer stem cells through Wnt/β-catenin signaling pathway. Mechanistically, AC104041 acts as ceRNA for miR-6817-3p, inducing Wnt2B ligand stabilization and β-catenin activation allowing HNSCC cells proliferation and metastasis [151, 152].

Moreover, significant decrease of MALAT1 expression levels using ASO-conjugated nanoparticles, reduces lung cancer cells migration in vitro and metastatic tumor nodule formation in vivo [153].

Furthermore, elevated levels of LINC00680 in esophageal squamous cell carcinoma (ESCC) were associated with large tumor size, advanced tumor stage, and poor prognosis. Mechanistic investigation revealed that LINC00680 sponging miR-423-5p thus regulating the oncogene p21-activated kinase 6 (PAK6) expression in ESCC cells. ASOs targeting LINC00680 inhibit ESCC cells proliferation, migration and invasion in vitro and ESCC tumor formation in vivo [154].

Despite the use of different approaches to regulate lncRNAs expression pattern, lncRNAs can be used as therapeutic molecules to target EMT [155]. EMT-inducer SNAIL TF requires lncRNA HOTAIR to recruits EZH2 to its epithelial target genes to repress their expression. An approach was recently designed to counteract lncRNA HOTAIR-associated EMT that was based on the use of a deletion-mutant form of the lncRNA HOTAIR (HOTAIR-sbid). HOTAIR-sbid contains the TF SNAIL-binding domain but the EZH2-binding domain is absent. Mechanistically, HOTAIR-sbid binds to SNAIL but is unable to mediate the interaction between the SNAIL and the histone methyl transferase EZH2. This, in turn, reduces the H3K27me3/EZH2-mediated repression of epithelial SNAIL-target genes. HOTAIR-sbid expression impairs HCC cellular motility, invasiveness, anchorage-independent growth, and responsiveness to TGFβ-induced EMT [156].

### Conclusion and perspectives

Metastasis is a multi-step process and considered a turning point in the fate of cancer progression and with regard to clinical outcome. Numerous factors and signaling pathways play an essential role in the metastasis of cancer cells in order to enhance their migration and invasion ability. EMT is the main feature of cancer cells metastasis and occurs through cytoskeleton remodeling, and their interaction with TME niche, including its cellular and non-cellular components.

Many studies showed that altered expression of lncRNAs correlated with cancer metastasis and poor clinical outcome. As with other hallmarks of cancer, lncRNAs regulate cancer cells metastasis through different signaling pathways, and metastasis-associated genes on both transcriptional and post-transcriptional levels by acting as guide/decoy for chromatin-modifying complexes or as ceRNA for anti-metastatic miRNAs.

Diverse approaches, including RNAi, ASOs and CRISPR-based methods, have yielded plentiful information about lncRNA functions and underlying mechanisms. Among these, genome-wide screening of lncRNA using pooled gRNA CRISPR/Cas9 approach to identify numerous cancer-associated lncRNAs altogether have proven itself a very powerful tool.

All the data generated so far about lncRNAs involvement in metastasis-associated pathways have brought quite valuable information to advance our knowledge about the topic. However, the transformation of this accumulated data into clinically useable information has not been yet achieved, neither as therapeutic tools nor as biomarkers predictive of clinical outcome. While the development of RNA-based therapeutics in the future may enable the targeting of lncRNAs, the main issue to be solved in our opinion would be the choice of target lncRNAs, reflected by the difficulties to define reliable lncRNA-based signature of clinical characteristics. These difficulties are inherent with the modes of actions of lncRNAs. Firstly, any ceRNA mode of action is dependent on the expression levels of the partner miRNA and targeted mRNAs. The relative amounts of each of these partners, as described in cell lines studies, might not reflect the overall clinical setting, and possibly impair the validity of predictive signatures or of potential therapeutic targets. Second, because of their ability to act through different mechanisms of action, the potential for off-target effects in vivo for lncRNA-based therapeutics appears quite high.

The recent development of patient-derived tumor organoids (PDTO) model systems may allow circumventing some of these problems. Compared to classical cancer cell lines, they have been shown to match with their tumor-of-origin both at the phenotypical and molecular level, and faithfully match patient’s response when exposed to drugs [157, 158]. Moreover, these models allow the study of invasion-related phenotypes when grown in the appropriate matrixes [159]. Interestingly, recent studies managed to establish organoid models derived from circulating colorectal cancer cells. The resulting model did reflect the molecular and phenotypical characteristics of the circulating cancer cells, including their hybrid EMT state [160]. Organoid models thus appear as best suited than classical cell lines to identify lncRNAs of interest, as potential biomarkers or therapeutic targets of genuine clinical relevance. Further, in this regard circulating tumor cells-derived organoid should of special interest in the context of metastasis-associated characteristics.

Moreover, beyond the direct clinical relevance of modulating cytoskeleton or ECM related pathways as a mean to counter metastasis and cancer aggressiveness, the connections between microenvironment and ECM with immune signaling might offer an alternative way to predict or orientate the response to immunomodulatory drugs targeting PD-1/L1 or CTLA4 [161, 162]. Whether this can be achieved with direct intervention on lncRNAs involved in these processes or by acting on the downstream determinants of their action by more usual approach with pharmacological inhibitors.

To conclude, while much has been done to study the roles and function of lncRNAs in metastasis-associated pathways in cancer, much more remains to be done. A more intensive use of genome wide screens for instance could help specify the most prominent lncRNAs in an extended repertoire of cancer types and/or model systems. In addition, the use of the most recent model systems for the study of cancer mechanisms and therapeutics would surely help in evidencing clinically-relevant lncRNAs modes of actions, and thus pave the way for the design of future therapeutic options.

## Data Availability

Not applicable.

## Abbreviations

ECM: Extracellular matrix
EMT: Epithelial-mesenchymal transition
TGF-β: Transforming growth factor-β
RTK: Receptor tyrosine kinase
LncRNA: Long non-coding RNA
PRC2: Polycomb repressive complex2
EZH2: Enhancer of zeste homolog 2
MLL: Mixed-lineage leukemia
TFs: Transcription factors
Erna: Enhancer RNA
SF: Splicing factor
ceRNA: Competing endogenous RNA
RISC: RNA-induced silencing complex
E-cadherin: Epithelial cadherin
N-cadherin: Neural cadherin
MMPs: Matrix metalloproteinases
ZEB: Zinc-finger E-box-binding
OC: Ovarian cancer
PTAR: Pro-transition associated RNA
KD: Knockdown
LC: Lung cancer
PCa: Pancreatic cancer
HCC: Hepatocellular carcinoma
SOX4: SRY-related HMG-box 4
ATB: Activated by TGF-β
BC: Breast cancer
GC: Gastric cancer
CRC: Colorectal cancer
MALAT1: Metastasis-associated lung adenocarcinoma transcript 1
G: Globular
F: Filamentous
CFL1: Cofilin
Rho: Ras homology
Cdc42: Cell division cycle 42
ROCK: Rho-associated coiled-coil containing protein serine/threonine kinase
LCAT1: Lung cancer associated transcript 1
DANCR: Differentiation antagonizing nonprotein coding RNA
LIMK1: LIM domain kinase 1
ZFAS1: ZNFX1 antisense RNA 1
PAK1: P21-activated kinase 1
TME: Tumor microenvironment
CAFs: Cancer-associated fibroblasts
Col-1: Collagen type (I)
HOTAIR: HOX transcript antisense RNA
NSCLC: Non-small cell lung cancer
RNAi: RNA interference
ASOs: Antisense oligo nucleotides
sgRNA: Single guide RNA
DSB: Double strand break
HR: Homologous recombination
NHEJ: Non-homologous end joining
dCas9: Dead Cas9
KRAB: Krüppel-associated box
HNSCC: Head and neck squamous carcinoma
ESCC: Esophageal squamous cell carcinoma
